# African swine fever: A re-emerging viral disease threatening the global pig industry

**DOI:** 10.1016/j.tvjl.2017.12.025

**Published:** 2018-03

**Authors:** P.J. Sánchez-Cordón, M. Montoya, A.L. Reis, L.K. Dixon

**Affiliations:** The Pirbright Institute, Pirbright, Woking, Surrey GU24 0NF, UK

**Keywords:** African swine fever, Control, Epidemiology, Immune responses, Pathogenesis, Vaccination

## Abstract

African swine fever (ASF) recently has spread beyond sub-Saharan Africa to the Trans-Caucasus region, parts of the Russian Federation and Eastern Europe. In this new epidemiological scenario, the disease has similarities, but also important differences, compared to the situation in Africa, including the substantial involvement of wild boar. A better understanding of this new situation will enable better control and prevent further spread of disease. In this article, these different scenarios are compared, and recent information on the pathogenesis of ASF virus strains, the immune response to infection and prospects for developing vaccines is presented. Knowledge gaps and the prospects for future control are discussed.

## Highlights

• Genotype II African swine fever virus (ASFV) circulating in Europe has high pathogenicity for domestic pigs and wild boar.
• Genotype II ASFV strains in Europe do not exhibit attenuation and have limited ability to establish persistent infections.
• The wild boar population in Eastern Europe plays an important role in the maintenance and spread of ASFV.
• Immune responses against ASFV are not fully understood, hampering the rational design of vaccines.
• Control relies on ‘stamping-out’ policies and control of pig movements, which have a significant impact on affected regions.

## Introduction

African swine fever (ASF) was first identified in East Africa in the early 1900s as a disease causing high mortality in domestic pigs (*Sus scrofa domesticus*). It was quickly established that warthogs (*Phacochoerus africanus*) could be a source of infection (Montgomery, 1921) and that this host, along with a species of soft ticks (*Ornithodoros* spp.) which live in warthog burrows, could be persistently infected with ASF virus (ASFV) without showing signs of disease (Plowright et al., 1969). ASFV is widely distributed in this ancient wildlife cycle in East Africa. Subsequently ASFV has spread in domestic pig populations throughout most sub-Saharan African countries. Transcontinental spread first occurred to Europe (Spain and Portugal) in 1957 and 1960, and from there to other European countries, South America and the Caribbean. With the exception of Sardinia, disease was eradicated from outside Africa in the mid-1990s. A second transcontinental spread to Georgia in the Caucasus occurred in 2007, with subsequent spread to neighbouring countries and further into Eastern Europe. It is now recognised that wild boar (*Sus scrofa*) have an important role in the spread and maintenance of ASFV in these regions.

Currently, ASFV is present in the Trans-Caucasus, parts of the Russian Federation and neighbouring countries, including Ukraine, Poland, Latvia, Lithuania, Estonia and Moldova, as well as, more recently, the Czech Republic and Romania. Outbreaks in domestic pigs have also occurred further east in the Russian Federation, including a region close to the Mongolian borders.^2^ The latter constitutes one of the most important jumps of the disease so far described. Further spread seems likely, since attempts to control the disease have not been effective.

## African swine fever virus

ASFV is a large DNA virus that replicates in the cytoplasm and is the only member of the *Asfarviridae* family. The virus encodes 150–165 proteins, which have ‘essential’ functions in virus replication, as well as ‘non-essential’ roles in host interactions, including evasion of host defences; for example, many proteins inhibit the early innate responses, including type I interferon and cell death pathways (Dixon et al., 2013).

Sequencing of the gene encoding the major capsid protein (B646L/p72) has defined 23 different genotypes of ASFV (Bastos et al., 2003, Achenbach et al., 2016). Genotype I is spread through West and Central Africa, was introduced to Europe in 1957 and 1960, and is currently in Sardinia. Genotype II was introduced to Georgia in 2007 and has spread through the Russian Federation and Eastern Europe (Rowlands et al., 2008, Gallardo et al., 2014). Sequencing has demonstrated that isolates circulating in Eastern Europe from 2007 to 2011 were almost identical (Malogolovkin et al., 2012). However, sequencing of ASFV isolates from wild boar found dead in Lithuania and Poland in 2014 identified minor variants that were identical to isolates obtained from Belarus in 2013, but different from isolates obtained from Russia in 2012 and Georgia in 2007 (Gallardo et al., 2014, Goller et al., 2015). In experimental and field samples from the Russian Federation, 3.7% of serum samples from wild boar were positive for antibodies against ASFV, indicating that few pigs survived infection, thus confirming the high virulence of circulating isolates (Mur et al., 2016).

## Control and economic impacts of African swine fever virus

Although ASF was first described almost a century ago, controlling the disease has proven to be a challenge, in particular because no vaccine is available. Following introduction to ASFV-free countries, the only control measures available are strict quarantine and biosecurity, animal movement restrictions and slaughtering affected/exposed animals.

**Fig. 1:**
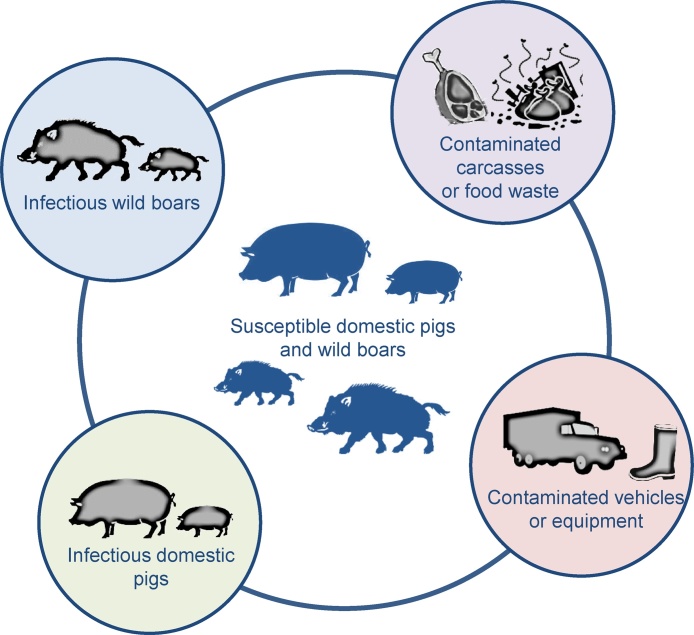
Potential transmission routes for African swine fever virus (ASFV) in Europe. Sources of infection include infectious domestic pigs (*Sus scrofa domesticus*) and wild boar (*Sus scrofa*), contaminated carcasses, food waste and contaminated vehicles or equipment. Soft ticks of *Ornithodoros* spp. have not been shown to be involved in transmission of ASFV in Eastern Europe, Russia or the Trans-Caucasus region. Wild boar are not present in Africa, but wild suids, including warthogs (*Phacochoerus africanus*) and bush pigs (*Potamochoerus larvatus*) can be persistently infected and act as a source of infection. *Ornithodoros* spp. ticks inhabiting warthog burrows or pig housing can also be involved in transmission in East Africa.

Measures to mitigate the risk of spread of ASFV in different commercial pig production systems and backyard holdings have been analysed by Bellini et al. (2016). The role of wild boar in the spread and maintenance of ASFV also has to be taken into consideration; for example, from 2015 to 2017, nearly 8000 ASFV positive wild boar were identified in Poland and the Baltic countries.^3^ The large amount of virus shed during the infectious period of the disease (Gabriel et al., 2011, Blome et al., 2012) and the ability of ASFV to survive for long periods in a protein rich environment, even under adverse conditions (Penrith and Vosloo, 2009, Costard et al., 2013), mean that infectious live wild boar and their carcasses should be considered in any control measures. The EU Commission has recommended procedures for control of ASF in domestic pigs and wild boar populations^4^; this includes reporting all dead wild boar found and testing of those hunted in control zones. Fig. 1 summarises methods of ASF transmission in Eastern Europe and the Russian Federation. The availability of a vaccine in the future could complement these control measures.

The high lethality of ASFV in domestic pigs, the introduction of massive culling campaigns and pig movement restrictions all contribute to the high socio-economic impact of ASF on pig production, global trade and people’s livelihoods. The impact is often greatest for resource poor livestock farmers in developing countries, who rely on pigs as an additional source of income and a relatively cheap source of protein.

In Eastern Europe and the Russian Federation, the greatest numbers of outbreaks in domestic pigs have been on small scale or backyard farms, which generally have lower biosecurity standards and, in some regions, have closer contact with infected wild boar. From 2014 to August 2017, the highest numbers of farms with ASF outbreaks were in Russia and the Ukraine, and relatively few notifications of ASF were in wild boar in these countries. From 2014 to 2016, most detections in Poland were in wild boar, but in 2017 there were more notifications in domestic pigs on commercial farms, with more than 90 farms infected. In contrast, in the Baltic States, relatively few farms have been affected (approximately 60 in total) and most notifications have been in wild boar. Relatively few large farms have been infected, but such cases result in the death or destruction of substantially larger numbers of pigs. From 2014 to 2017, almost 800,000 pigs have died or been destroyed as a result of ASF in Eastern Europe and the Russian Federation. ASFV infection in wild boar can have an impact on the hunting industry, but perhaps the greatest impact is the threat and economic impact on commercial pig farms.

It is difficult to produce overall figures on the economic costs of ASF and thus estimates can vary substantially. As a result of outbreaks of ASF in 2014 and 2015 in Poland, Lithuania, Latvia and Estonia, the value of exports of pork and pork products was reduced by US$961 million,^5^ representing up to 50% of exports.^6^ Introduction of ASFV into Denmark could result in losses of US$12 million in direct costs and US$349 million in exports (Halasa et al., 2016). In Russia, ASF was estimated to have cost US$267 million in 2011.^7^ Further spread of ASF to China could have disastrous consequences, recognising that China contains more than half of the world’s pig population.

## Pathogenesis and transmission of natural African swine fever virus infections

Different courses of ASF have been described in domestic pigs in the field, historically from Spain and Portugal (Gomez-Villamandos et al., 2013). Disease manifestations include peracute, acute, subacute and chronic forms. In the peracute form, pigs die within 4 days post-infection (DPI) without gross lesions. The acute form can result in the death of a high proportion of infected pigs (90–100% mortality) 4–21 DPI, with characteristic pathological changes due to vasculitis, including skin erythema, pulmonary oedema, hyperaemic splenomegaly, haemorrhagic lymphadenitis, and petechial haemorrhages in the lungs, urinary bladder and kidneys. In the subacute form, which is caused by moderately virulent isolates, the mortality is 30–70%, incubation periods are longer (pigs die after 20 DPI) and clinical signs tend to be less marked; however, vascular changes, mainly haemorrhage and oedema, are more severe than those reported in the acute form. Low virulence isolates can cause chronic forms of the disease, which are characterised by the absence of vascular lesions and low mortality rates, but signs such as delayed growth, emaciation, joint swelling, skin ulcers and lesions associated with secondary bacterial infection (Sánchez-Vizcaíno et al., 2015).

The existence of sub-clinical or inapparent infections has also been suggested in survivor pigs, which are infected but do not display clinical signs or the lesions described in chronic disease. Virus can persist for prolonged periods in tissues or blood from recovered pigs or following infection with low virulence isolates, which might contribute to virus transmission, disease persistence, sporadic outbreaks and ASFV introduction into disease-free zones (Penrith and Vosloo, 2009, Costard et al., 2013, Gallardo et al., 2015). Recent studies in Africa have identified ASFV sequences in apparently healthy pigs in Uganda (Kalenzi Atuhaire et al., 2013) and Kenya (Thomas et al., 2016), suggesting that reduced virulence isolates may be circulating in these regions. There is limited experimental evidence for transmission from persistently infected to naïve animals. The relevance of carrier animals in the field is not clear.

A potential role of carrier domestic pigs as a source of infection has been suggested in Kenya and Uganda, in which healthy pigs can be positive for ASFV by PCR in tissues, but negative for ASFV in blood by PCR and serology (Okoth et al., 2013, Abworo et al., 2017). Such carriers may play a role in maintenance of ASFV in pig production systems where persistent clinical ASF outbreaks regularly occur. A high level of co-infections with other pathogens has been suggested as a factor that could influence latency and shedding of ASFV (Abworo et al., 2017).

Following the introduction of ASFV genotype II to the Trans-Caucasus and Russian Federation in 2007, most reports have been of the acute form of disease with high lethality in domestic pigs and wild boar. In the Russian Federation from 2007 to 2014, epidemics of ASF in wild boar and domestic pigs were independent from each other in the Krasnodar region, whereas there were regular spill-overs from domestic pigs to wild boar in the Tver region (Vergne et al., 2017). In contrast, in the Baltic States, ASFV spread extensively in wild boar, with only occasional spill-over to domestic pigs. In Estonia, there is evidence for two separate introductions of virus into wild boar during the study period. In the Southern region, disease typical of acute ASF was observed; dead wild boar were virus positive and antibody negative. In contrast, clinically healthy antibody positive wild boar were identified in the North-Eastern region. One possible explanation for these differences is that a moderately virulent isolate is circulating in this region and a greater number of animals survive (Nurmoja et al., 2017).

The dramatic spread of ASFV in wild boar in the Baltic States is unprecedented and the transmission mechanisms involved remain unclear, but may include the movement of infected wild boar, infected wild boar carcasses and/or environmental contamination. A role for persistently infected carrier animals has also been suggested. Spread in domestic pigs may result from spill-over infections from wild boar or through the well-established routes involving movement of pigs, pork or infected fomites (Guinat et al., 2016).

## Pathogenesis of experimental Eastern European isolates of genotype II African swine fever virus

Transmission from persistently infected carrier pigs has been postulated to have a role in maintaining long term ASFV infection in regions where disease has become endemic (Sánchez-Vizcaíno et al., 2012). Long term persistence following infection of pigs with genotype I isolates of reduced virulence has been demonstrated (Wilkinson, 1984, Carrillo et al., 1994). Experimental infections have demonstrated transmission from pigs persistently infected with the low virulence genotype I NH/P68 isolate to in-contact pigs (Gallardo et al., 2015).

Several studies have investigated the virulence of currently circulating ASFV genotype II isolates in Europe. Although disease caused by ASFV isolates of high and moderate virulence have been widely studied since the disease first appeared in the Iberian Peninsula (Gomez-Villamandos et al., 2013), little is known about ASF in wild boar, despite their importance in disease spread, their high susceptibility to disease and their possible role as virus carriers in the current European scenario (Blome et al., 2013). Oral and intramuscular experimental infections of piglets, juveniles and adult wild boar with moderate and high doses of genotype II isolates from the Caucasus region (Armenia in 2008 and Chechen Republic in 2009) resulted in an acute form of ASF with 100% lethality within less than 12 days (Gabriel et al., 2011, Blome et al., 2012). No antibodies were detected in serum samples (Blome et al., 2012). Although shedding of ASFV through nasal discharge or faeces seemed to be limited, virus transmission to domestic pigs and wild boar used as in-contact controls was effective, also inducing acute disease in these recipients. Blood was shown to be highly infectious and is likely to be the main source of infection for in-contact pigs (Gabriel et al., 2011).

In another study with a similar ASFV strain (Georgia 2007/1), intramuscular inoculation of domestic pigs with 10^2^ 50% haemadsorbing doses (HAD_50_) and in-contact transmission resulted in development of acute disease in all pigs (Guinat et al., 2014). One inoculated pig survived until day 12, whereas all other inoculated pigs were euthanased by day 9, while all in-contact pigs were euthanased at 14–18 days post-exposure (DPE). Only the inoculated animal that survived until day 12 post-infection had detectable ASFV-specific antibodies. ASFV was excreted at low levels through the nasal and rectal routes. This study suggested that genotype II isolates from the early phase of the epidemic (2007–2009) had a limited potential to cause persistent infection.

The incubation period, period of latency and infectious periods in the course of ASFV infection are related to the quantity of virus shedding and route of virus excretion, which may in turn be related to the infectious dose and the virus isolate (Howey et al., 2013). The possibility that low dose genotype II ASFV infections might lead to prolonged incubation times and chronic disease, or the development of a carrier state, was investigated by Pietschmann et al. (2015). Low-dose oronasal infections of domestic pigs and European wild boar were undertaken with the Armenia 08 isolate. Very low doses of this isolate (3 or 25 haemagglutinating units, HAU) were sufficient to infect especially weak or runted animals by the oronasal route. The time to onset of clinical signs was delayed in these low dose infections, but there were no changes in the course and outcome of infection; infected pigs developed acute and subacute forms of ASF with severe vascular changes after incubation periods of 11 days to 4 weeks (Pietschmann et al., 2015).

Intramuscular infections of domestic pigs with a low dose (10 HAD_50_) of a Lithuanian isolate (LT14/1490) from 2014 caused an acute fatal form of ASF, requiring euthanasia of 7/8 pigs at 7–9 DPI (Gallardo et al., 2017). One directly inoculated pig had a delayed onset of clinical signs similar to that of in-contact pigs; this pig may have been infected by contact with other infected pigs. All but one of the in-contact pigs were euthanased at 14–22 DPE with signs typical of acute ASF. One in-contact pig remained unaffected, with very low and intermittent levels of sequences being detected until the end of the experiment at 61 DPE; no antibody response was detected and low levels of virus sequences were detected in tissues samples.

Experimental intranasal and intramuscular inoculations of domestic pigs with low (50 HAD_50_) and high (5000 HAD_50_) doses of Russian ASFV isolates collected in 2013 from domestic pigs and wild boar induced acute forms of ASFV in both inoculated and in-contact pigs (Vlasova et al., 2015). Incubation and infectious periods were shortest with high doses inoculated by the intramuscular route, and longest with low doses administered by the intranasal route (Vlasova et al., 2015). Antibodies against ASFV were detected in 3.7% serum samples from this experiment (Mur et al., 2016).

Nurmoja et al. (2017) isolated ASFV by animal passage from PCR positive tissue samples from a wild boar outbreak with apparently low mortality in North-Eastern Estonia in 2015. In European wild boar inoculated by the oronasal route with 10^4.5^ HAU ASFV, all but one pig developed the severe acute form of ASF at 7–13 DPI; the survivor had high antibody levels, indicating that virus had replicated. However, no transmission occurred from the survivor to in-contact pigs when they were commingled from 50 to 96 DPI. These results suggest that not all surviving pigs become carriers of ASFV.

Despite the low mortality rates suggested by some field observations, for example in Estonia (Khomenko et al., 2013, Nurmoja et al., 2017), and the existence of genetic variants of ASFV (Chapman et al., 2011, Gallardo et al., 2014), so far experimental studies using different genotype II ASFV isolates from different regions of Eastern Europe and the EU have not demonstrated clear evidence for reduced virulence in either wild boar or domestic pigs. In most of cases, a detectable antibody response was not observed due to the rapid onset of acute disease. Despite descriptions of occasional survivors (Gallardo et al., 2017, Nurmoja et al., 2017), most of the experiments have demonstrated a limited potential of genotype II ASFV isolates to cause persistent infection and thus generate ASFV carriers. However, continued monitoring of the virulence of circulating isolates is required and there is a need to evaluate the mechanisms by which ASFV is maintained within wild boar populations. A study in which the behaviour of free ranging wild boar towards the carcasses of dead conspecifics was monitored, there was no evidence of cannibalism (Probst et al., 2017). However, this study also demonstrated that carcasses can be contaminated with ASFV for a considerable length of time (Probst et al., 2017).

## Development of vaccines against African swine fever virus

Vaccination is one of the best control measures for infectious diseases; however, development of vaccines against ASFV has been deterred by large gaps in our knowledge of ASFV infection and immunity. So far, the precise nature of the protective responses has not been determined and protective antigens have not yet been identified, hampering the rational design of vaccines. In addition, mechanisms by which the virus modulates the host response to infection are poorly understood. Once protective antigens have been identified, broadly effective and cross-protective rational vaccines against relevant ASFV strains could be developed (Table 1).

**Table 1:** Summary of approaches for African swine fever virus (ASFV) vaccine development.

Vaccine approach	Advantages	Disadvantages	Comments
Subunit vaccines (recombinant proteins, DNA vaccines, virus vector)	Safety	Requirement for boost likely	Partial protection achieved with recombinant proteins or by DNA vaccination
	DIVA compatibility	Knowledge of protective antigens required	
	Established scale up method		
	High containment not required for production		
Inactivated virus	Safety	Mainly stimulates antibody response	Not effective for ASFV
Live attenuated vaccines	Stimulates both cellular and antibody responses	Safety issues relating to adverse post-vaccination reactions and virus persistence	Natural attenuated and gene deleted viruses tested
	Single dose may induce long term immunity	Production requires high containment and is virus specific	Optimised combinations of gene deletions required to achieve acceptable levels of safety and efficacy
	Knowledge of protective antigens not required	DIVA compatibility more difficult	Cell culture methods for commercial production needed
	High efficacy can be achieved		

DIVA, Differentiating infected from vaccinated animals.

Generally, vaccines against viruses have been developed using viral inactivation, attenuation or by generating subunit vaccines. In the case of ASFV, traditional inactivated vaccines were shown to be unsuccessful more than 40 years ago (Stone and Hess, 1967). Similarly, recent re-assessment of inactivated ASFV preparations combined with state-of-the-art adjuvants have confirmed that binary ethyleneimine (BEI)-inactivated ASFV vaccines did not induce protection after challenge (Blome et al., 2014). Therefore, inactivation does not appear to be an effective strategy for ASFV vaccine development.

Live-attenuated vaccines can induce strong and long-lasting immune responses. ASFV attenuation was initially performed by sequential passage in cell culture; these attenuated viruses induced protection in pigs after challenge with the homologous virulent parental strain (Stone et al., 1968). Immunisation of pigs with another non-virulent naturally attenuated genotype I OUR T88/3 isolate of ASFV induced protection against different isolates belonging to the same genotype (Boinas et al., 2004, Oura et al., 2005, King et al., 2011, Abrams et al., 2013, Mulumba-Mfumu et al., 2016, Sánchez-Cordón et al., 2017a), as well as cross-protection against isolates belonging to other genotypes (King et al., 2011).

A new generation of ASFV gene-deletion mutants have been generated that have induced protective responses in pigs (O’Donnell et al., 2015a, Reis et al., 2016, O’Donnell et al., 2017, Monteagudo et al., 2017, Sánchez-Cordón et al., 2017b). Cross-protection against a genotype II isolate was induced by a genotype I attenuated virus, suggesting that cross-protective attenuated virus vaccines may be feasible. A mutant ASFV with deletion of the *DP148R* gene had reduced virulence in pigs and induce high levels of protection against challenge, even though virus replication in culture was not reduced (Reis et al., 2017a). However, further investigations are required to define combinations of virulence genes to achieve acceptable levels of safety and efficacy in different ASFV strains.

Generation of subunit vaccines is possible when certain viral protein(s) have been identified that confer full protection and when the optimal delivery vector/conditions for immunisation are identified. In the case of ASFV, both concepts are far from established. Early vaccination studies with B cell immunodominant ASFV subunit recombinant proteins (p30, p54 and p72) had variable success (Gomez-Puertas et al., 1998, Barderas et al., 2001, Argilaguet et al., 2011, Argilaguet et al., 2012). An ASFV expression library containing approximately 4000 individual plasmid clones (excluding p30, p54 and CD2v) showed some correlation between protection and CD8^+^ T cell responses (Lacasta et al., 2014).

Robust cellular and humoral responses have been elicited after immunisation of pigs using a cocktail of adenovirus-vectored ASFV with selected antigens (Lokhandwala et al., 2016) or using a bioinformatics approach for vaccine target prediction (Lopera-Madrid et al., 2017). The relevance of these induced immune responses to protection against ASFV requires further evaluation in challenge studies.

## Immune responses correlating with protection against African swine fever virus

Considering the risk and importance of ASF, it is surprising that protective immune mechanisms against ASFV are far from understood. Under experimental conditions, older pigs appear to have better survival than younger pigs after infection with a moderately virulent isolate of ASFV, regardless of the inoculation dose (Post et al., 2017). ASFV mainly replicates in myeloid lineage cells, especially antigen presenting cells (APCs) such as monocytes/macrophages and dendritic cells (Malmquist and Hay, 1960, Wardley and Wilkinson, 1977, Gonzalez-Juarrero et al., 1992b, Carrillo et al., 1994, Gregg et al., 1995a, Gregg et al., 1995b, Sánchez-Cordón et al., 2008). There is evidence of modulation of major histocompatibility class II expression in the spleen of pigs infected with a virulent strain of ASFV (Gonzalez-Juarrero et al., 1992a). Differences in cytokine secretion following infection of monocytes and macrophages have been reported following infection with attenuated and virulent ASFV (Franzoni et al., 2017). The mechanisms whereby ASFV overcomes the barriers to replication in macrophages inhibiting host defence pathways have been reviewed by Reis et al. (2017b).

Type I interferon (IFN) is a crucial component of the innate response to viral infection (Goodbourn and Randall, 2009) and ASFV has developed strategies to counteract this response. Non-virulent/low virulence ASFV strains, which lack genes from MGF360 and MGF505, induce the expression of IFN during infection of macrophages in vitro, whereas virulent ASFV strains do not (Afonso et al., 2004, Zhang et al., 2006, Gil et al., 2008). However, IFNα and IFNβ have been detected in the serum of pigs infected with virulent Georgia 2007/1 (Karalyan et al., 2012), suggesting that virulent ASFV induces IFN in vivo. The early induction of type I IFN in infected pigs is probably critical for control of infection. In vitro infection of porcine leucocytes enriched for dendritic cells with the low virulence isolate OUR T88/3 (which lacks MGF360 and MGF505), induced high levels of interferon (Golding et al., 2016). Deletion of MGF360 and MGF505 increased the sensitivity of virus replication to interferon pre-treatment, suggesting that the proteins encoded by these regions have a role in inhibiting IFN responses or the antiviral state, as well as IFN induction (Golding et al., 2016).

ASFV encodes anti-apoptotic proteins, including A179L (a Bcl-2 family member), A224L (a member of the family of ‘inhibitor of apoptosis proteins’, IAP), EP153R (a C-type lectin) and DP71L (which inhibits activation of the stress activated pro-apoptotic pathways) (Dixon et al., 2017).

Neutralising antibodies are often important for protection against virus infections. However, data on the role of humoral responses against ASFV are controversial. Some reports underline the role of specific antibodies, since antibody transfer through colostrum conferred a degree of protection in suckling pigs (Schlafer et al., 1984) and passively transferred antibodies against ASFV protected pigs from lethal infection (Onisk et al., 1994, Escribano et al., 2013). Immunisation of pigs with recombinant p30 and p54 proteins induced the production of neutralising antibodies and conferred protection in 50% of infected pigs (Gomez-Puertas et al., 1998). However, in a study by Neilan et al. (2004), pigs immunised with baculovirus-expressed p30, p54 and p72 were not protected against ASFV challenge, despite the induction of neutralising antibodies. Partial protection against ASFV was induced by immunisation with an ASFV expression library in the absence of detectable antibodies (Lacasta et al., 2014). Data compiled from vaccination/challenge experiments in pigs indicate that protective immunity against ASFV was haemadsorption inhibition serotype-specific (Burmakina et al., 2016). These results suggest that antibodies alone are not sufficient to induce protective immunity against ASFV.

Cellular immune responses (particularly CD8α^+^ cells) have been identified as an important protective mechanism when attenuated ASFV strains were used for immunisation. Depletion of CD8α^+^ cells prevents protection in challenged animals previously vaccinated with the attenuated ASFV strain OURT88/3 (Oura et al., 2005, Takamatsu et al., 2013). In addition to cytotoxic T cells, CD8α is expressed on natural killer (NK) cells, NK T cells, subsets of γd T cells and memory helper T cells (Denyer et al., 2006, Gerner et al., 2009, Gerner et al., 2015). The roles of these subsets in immune responses against ASFV have not been characterised fully, although a correlation between γδ T cells and survival has been reported (Post et al., 2017).

Indirect evidence of the involvement of cellular immunity in protection against ASFV also comes from vaccination with DNA plasmids and baculovirus vectors encoding ASFV fusion proteins. These vaccines induced cytotoxic T lymphocyte responses and conferred partial protection against ASFV challenge (Argilaguet et al., 2012, Argilaguet et al., 2013). CD8^high^ T cell in vitro proliferation responses have been correlated with protection against ASFV infection (Lacasta et al., 2015).

Cytokines and chemokines are important drivers of immune and inflammatory responses. However, the number of in vivo studies reported in the context of ASFV infection is limited. There is a correlation between IFNγ secreting cells (detected by ELISpot) and protection against ASFF (Alonso et al., 1997, King et al., 2011, Argilaguet et al., 2012, Argilaguet et al., 2013, Lacasta et al., 2014, O’Donnell et al., 2015b, Reis et al., 2016). However the nature of the cells secreting this cytokine is not known. Other cytokines, such as IL-10, have been related to lack of protection in the late stages of ASFV infection (Sánchez-Cordón et al., 2017a) and survival after infection (Post et al., 2017). In serum from pigs infected with the high virulence ASFV isolate Benin 97/1, levels of CCL2 protein were increased greater than 30 fold and CXCL10 protein was also detected (Fishbourne et al., 2013).

## Conclusions

The continued spread of ASFV in Africa and Europe demonstrates a potential for further spread in other regions of the world. There are still many gaps in our knowledge of the biology of ASFV, its interaction with hosts, immune responses correlating with protection and how these can be activated. Vaccine development against ASF has been hampered by large gaps in knowledge concerning ASFV infection and immunity. Current studies indicate that ASFV isolates circulating in Eastern Europe and the Russian Federation are highly virulent and kill most of the infected domestic pigs and wild boar. There is a need for continued monitoring in the field, along with experimental infections, to identify ASFV isolates of altered virulence. Increased use of complete virus genome sequencing may identify additional genetic markers that can be used to trace the spread of virus isolates, as well as to identify potential virulence markers. The main mechanisms of virus spread, particularly in wild boar in Europe, are not clear.

## Conflict of interest statement

None of the authors of this paper has a financial or personal relationship with other people or organisations that could inappropriately influence or bias the content of the paper.

## Footnotes

2 See: https://www.oie.int/wahis_2/public/wahid.php/Wahidhome/Home (accessed 1 January 2018).

3 See: https://www.efsa.europa.eu/en/events/event/151123 (accessed 1 January 2018).

4 See: https://ec.europa.eu/food/animals/animal-diseases/control-measures/asf_en (accessed 1 January 2018).

5 US$1.00 = ∼UK£0.74 = ∼€0.83 as at 31 December 2017.

6 See: https://gain.fas.usda.gov/Recent%20GAIN%20Publications/Expansion%20of%20African%20Swine%20Fever%20in%20Poland%20and%20Baltic%20Countries._Warsaw_Poland_11-7-2016.pdf (accessed 1 January 2018).

7 See: https://www.pig333.com/latest_swine_news/russia-economic-impact-of-african-swine-fever_5019/ (accessed 1 January 2018).
